# Novel method to achieve crystallinity of calcite by *Bacillus subtilis* in coupled and non-coupled calcium-carbon sources

**DOI:** 10.1186/s13568-020-01111-6

**Published:** 2020-09-29

**Authors:** Héctor Ferral-Pérez, Mónica Galicia-García, Bonifacio Alvarado-Tenorio, Aldo Izaguirre-Pompa, Marisela Aguirre-Ramírez

**Affiliations:** 1 Laboratorio de Biología Celular y Molecular, Departamento de Ciencias Químico-Biológicas, Instituto de Ciencias Biomédicas, Universidad Autónoma de Ciudad Juárez, 32310 Cd. Juárez, CHIH Mexico; 2 Laboratorio de Electroquímica, Departamento de Ciencias Químico-Biológicas, Instituto de Ciencias Biomédicas, Universidad Autónoma de Ciudad Juárez, 32310 Cd. Juárez, CHIH Mexico; 3 Laboratorio de Bioquímica Funcional y Proteómica del Estrés, Departamento de Ciencias Químico-Biológicas, Instituto de Ciencias Biomédicas, Universidad Autónoma de Ciudad Juárez, 32310 Cd. Juárez, CHIH Mexico; 4 Laboratorio de Geología, Departamento de Ingeniería Civil y Ambiental, Instituto de Ingeniería y Tecnología, Universidad Autónoma de Ciudad Juárez, Cd. Juárez, CHIH Mexico

**Keywords:** *Bacillus subtilis* organomineralization, Biogenic calcite, Calcium carbonate composites, Micrite

## Abstract

Bacteria mineralization is a promising biotechnological approach to apply in biomaterials development. In this investigation, we demonstrate that *Bacillus subtilis* 168 induces and influences CaCO_3_ composites precipitation. Crystals were formed in calcium-carbon non-coupled (glycerol + CaCl_2_, GLY; or glucose + CaCl_2_, GLC) and coupled (calcium lactate, LAC; or calcium acetate, ACE) agar-sources, only maintaining the same Ca^2+^ concentration. The mineralized colonies showed variations in morphology, size, and crystallinity form properties. The crystals presented spherulitic growth in all conditions, and botryoidal shapes in GLC one. Birefringence and diffraction patterns confirmed that all biogenic carbonate crystals (BCC) were organized as calcite. The CaCO_3_ in BCC was organized as calcite, amorphous calcium carbon (ACC) and organic matter (OM) of biofilm; all of them with relative abundance related to bacteria growth condition. BCC-GLY presented greatest OM composition, while BCC-ACE highest CaCO_3_ content. Nucleation mechanism and OM content impacted in BCC crystallinity.

## Key points

- *B. subtilis* mineralized colonies show spherulitic and botryoidal growth.
- BCC are mainly composed for calcite, ACC and CaCO_3_ precipitant wrapped up in EPS.
- Crystalitte is evident in non-coupled calcium-carbon sources conditions.

## Introduction

Bacterial CaCO_3_ mineralization is a phenomenon that occurs in sediments, caves, hot springs, soils and even in monuments and buildings (Ciferri et al. 2000; Rusznyák et al. 2012; Páramo et al. 2015). In nature, calcium carbonate biomineralization occurred in three different pathways: (i) controlled biological mineralization (CBM), (ii) induced biological mineralization (INDBM) and (iii) influenced biological mineralization (INFBM) (Dove et al. 2003). In CBM, organisms produce ordered mineral structures by a specified enzymatic mechanism encoded in their genes (Ngwenya 2016). Moreover, INDBM and INFBM are passive mechanisms, where physiological activity induces spontaneous precipitation of ions (Knorre and Krumbein 2000) or mineralization are influenced by extracellular polymeric substances (EPS) and biofilm geometry (Dupraz et al. 2009), respectively. CaCO_3_ precipitation is strongly influenced by basically four conditions: high pH environment (pK_2_ [CO] = 10.3 at 25 °C), oversaturation of Ca^2+^ and CO_3_^2−^, and availability of nucleation sites (Dupraz et al. 2009).

It has been demonstrated the occurrence of *B. subtilis* culture alkalinization in rich media (Robinson et al. 1991) probably due to spontaneous extracellular proteins, or amino acids deamination (Dupraz et al. 2009). Besides, when cells oxidate the carbon sources, the resulting product comprises CO_2_ and water. Under alkaline conditions, CO_2_ (pK_2_ [CO] = 10.3 at 25 °C) spontaneously evolve into CO_3_^2−^ (Stumm 1990). In addition, some *Bacillus* species could accelerate the hydration of CO_2_(g) through carbonic anhydrase activity (Dhami et al. 2014).

Additionally, calcium uptake and extrusion in bacteria are passive processes promoted by osmotic forces across a Ca^2+^/H^+^ antiporter protein. Besides, there is a Ca^+2^/Na^+^ antiporter pump that also maintains low intracellular calcium concentration (De Vrij et al. 1985) and, creates a high saturated microenvironment near to cell wall and EPS (Banks et al. 2010; Perito and Mastromei 2011; Meier et al. 2017).

Finally, *B. subtilis* produced CaCO_3_ minerals by INFBM; where EPS, membrane, and cell wall structures act as nucleation sites (Perito et al. 2014; Priya et al. 2016). Specifically, the metabolism of fatty acids plays a key role in biomineralization process (Perito et al. 2000; Barabesi et al. 2007), such as dipicolinic acid (Marvasi et al. 2017).

In vitro, *B. subtilis* promotes calcite crystals formation in presence of different calcium sources such as calcium lactate (Sierra-Beltran et al. 2014), calcium acetate (Shirakawa et al. 2011) or calcium chloride (Shirakawa et al. 2011; Micallef et al. 2016). In most of those cases, the addition of urea to promote pH increase is recurrent. Nevertheless, a recent work shows that non-ureolityc *Bacillus* strains that precipitates calcite could be used to mortar healing (Reeksting et al. 2020).

In order to propose an alternative white biotechnology method, this research demonstrated that *B. subtilis* facilitates CaCO_3_ precipitation through non-ureolytic pathway. As well, the acquiring of different crystallinity biogenic calcite could be achieved using either coupled or non-coupled calcium-carbon sources.

## Materials and methods

### Calcium carbonate biogenic crystal production and recovery

*Bacillus subtilis* 168 (ATCC^®^27370^TM^) strain was used in this work. Bacteria pre-culture was propagated in Nutrient broth (BD Bioxon, Cuatitlán Izcalli, México) by 24 h, at 37 °C. Petri dishes with of four different agar composition were inoculated with 1.17 × 10^7^ cells mL^−1^. The plates were incubated at 37 °C for 9 days. Nutrient Agar (BD Bioxon, Cuatitlán Izcalli, México) medium was supplemented with 0.026 M glycerol (Bio Basic, San Nicolas de la Garza, México) + 0.1 M CaCl_2_ (GLY), 0.013 M glucose (HYCEL, Zapopan, México) + 0.1 M CaCl_2_ (Jalmek, San Nicolás de la Garza, México) (GLC), 0.1 M calcium lactate (Cosmopólita, Naucalpan de Juárez, México) (LAC) or 0.1 M calcium acetate (LABESSA, Ciudad de México, México) (ACE). Bacteria growth modified pH values in liquid Nutrient Broth in all supplemented conditions. Initial pH varied between 6.5 and 7.1, and after eight hours, it increased until 9.3 as expected (Additional file 1: Fig. S1).

After incubation time, to detach the mineralized colonies from agar, culture was washed (López-Moreno et al. 2014) with 5 mL of boiling water, for calcium acetate and calcium lactate media, 2–3 washes were needed and for glucose and glycerol 4–6 washes were need because the producer amount of EPS. The water was recovered in a clean recipient to allow crystals sedimentation by 10 min. The crystals were washed with boiling water until the supernatant became clear. To eliminate the excess of organic residuals, sediment was washed several times with 1:3 acetone-alcohol (HYCEL, Zapopan, México) solution in vortex by 10 s, until supernatant became clear. The supernatant was thrown out and crystals were oven at 80 °C for 12 h to evaporate the rest of water and solvent.

### Petrographic analysis

A petrographic analysis was performed to internally characterize the crystals using polarizing microscopy. Standard petrographic thin section procedures (Murphy 1986) were modified to perform thin section of one fraction of BCC powder and flake aggregates. BCC were encapsulated into clear epoxy resin (COMEX, Mexico City, México) on the flat surface of a standard slide glass (26 × 46 mm). After curing epoxy resin, were trimmed and grinded until reach 100 µm thickness employing a saw/grinder machine (Ingram Thin Section Model 65). Encapsulated samples were finished by hand using silicon carbide and alumina abrasives in order to get ~ 25 µm of thickness and a polished surface. Thin sections were observed in Leica Petrographic Microscope DM2700 P with cross Nicols. The integrity of all samples was first evaluated in bright field (e.g. Additional file 1: Fig. S2).

### SEM analysis

Other fraction of BCC was sputtered with Pt/Ag layer by cathodic sputtering (MNT-JS1600, Micronano Tools) during 1 min (Folk and Lynch 1997). BCC were observed by scanning electron microscopy (SEM) in a JEOL JSM-7000F field emission scanning electron microscope. To determinate crystals elemental composition, energy dispersive x-ray spectroscopy (EDX) were performed.

### XRD analysis

Polymorphism and crystalline structures were determined by X-ray diffraction (XRD) using powder diffraction data method (Kontoyannis and Vagenas 2000). Panalytical X´pert Pro X-Ray MRD diffractometer was used; the X-ray emission was produced by copper cathode at Kα1 = 1.5405 Å wavelength and 20 to 80 2θ degrees by 0.001step protocol. The diffractograms were analyzed by means of the MATCH software.

### Crystallite size and crystallinity index determination

The theoretical crystallite size (τ) was calculated using the Scherrer equation (Patterson 1939; Eq. 1), using 0.9 as shape factor (K), the specific wavelength (λ) provide by the XRD equipment, the line broadening half the maximum intensity (β) and the Bragg angle (θ) of the more intense plane of the calcite (104) (Person et al. 1995; Merino and Morales 2008).

1 $$\tau = \frac{K \lambda }{\beta cos\theta }$$

Likewise, the area of the most intense peak (104) was determined, and compared with respect to mineral calcite, which has a crystallinity index (CI) of 99%. The area of (104), (006), (110) and (113) peak was calculated in order to calculate the crystalline index (IC; Eq. 2).

2 $$IC = \frac{{\mathop \sum \nolimits_{{}}^{{}} Area \;of\;peaks \;pattern}}{{\mathop \sum \nolimits_{{}}^{{}} Area\;of\;peaks\;sample}}$$

### ATR-FTIR analysis

The crystalline and amorphous phases composition was performed by Fourier Transformed Infra-Red spectrometry (ATR-FTIR). Crystals were analyzed in Bruker Alfa Platino-ATR spectrophotometer from 4000 to 400 nm.

### Thermal stability

To analyze crystal composition and thermal stability, 5 mg of biogenic crystals were compacted in aluminum pans. An SDT Q600 TA equipment was used for thermogravimetric analysis, temperature range was from 35 to 850 °C, heating rate was 10 °C min^−1^, working atmosphere was air at 50 mL min^−1^ of gas flow.

## Results

### Composites biomineralization

**Fig. 1:**
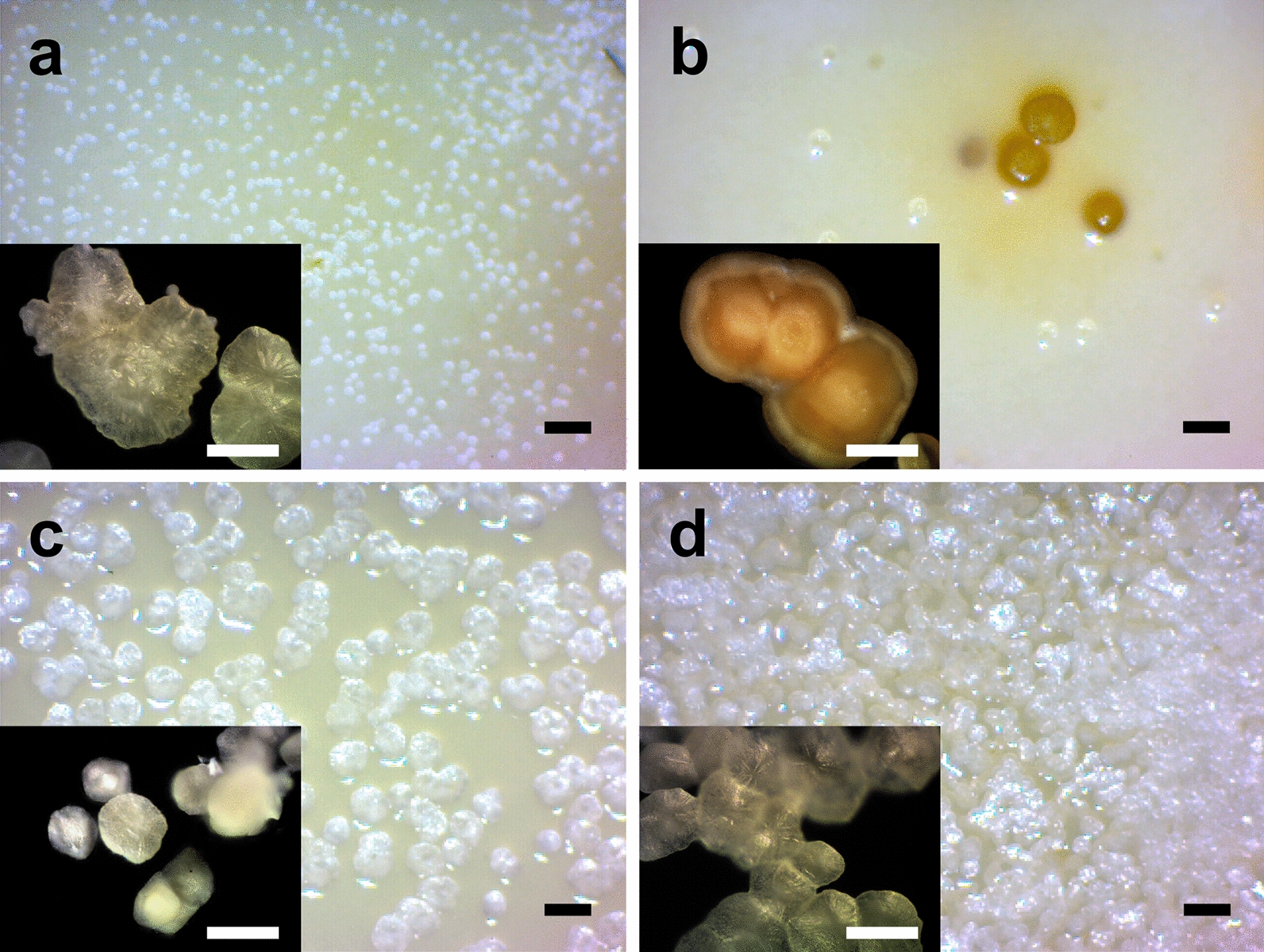
Cultures of *B. subtilis* and mineralized cell colonies formed in coupled and non-coupled calcium-carbon sources media. Images show stereoscopic view of plates and metallographic micrographs of recovered composites from **a** GLY, **b** GLC, **c** LAC, and **d** ACE media. Scale bars: 1 mm (black); 100 μm (white)

After 9 days of incubation at 37º C, *B. subtilis* mineralized colonies were harvested from Petri dishes with nutrient agar supplemented with glycerol + CaCl_2_ (GLY), glucose +CaCl_2_ (GLC), calcium lactate (LAC) or calcium acetate (ACE). Colonies formed in GLY and GLC (Fig. 1a, b) were scattered distributed over the plate comparatively of LAC or ACE (Fig. 1c, d) conditions. Once the material was detached with hot water (~ 90 °C), washed and cleaned up from non-mineralized cells and EPS, it was observed at metallographic microscope.

The bigger confluent colonies were obtained in both non-coupled conditions (GLY and GLC), but with irregular forms in GLY (Fig. 1a) than GLC (Fig. 1b); these were orange pigmented too. Coupled conditions showed smaller mineralized colonies, with typical smooth and regular borders; where the saturation of Petri dish was predominantly in ACE condition (Fig. 1d).

### Internal structures of biogenic calcium carbonate crystals

**Fig. 2:**
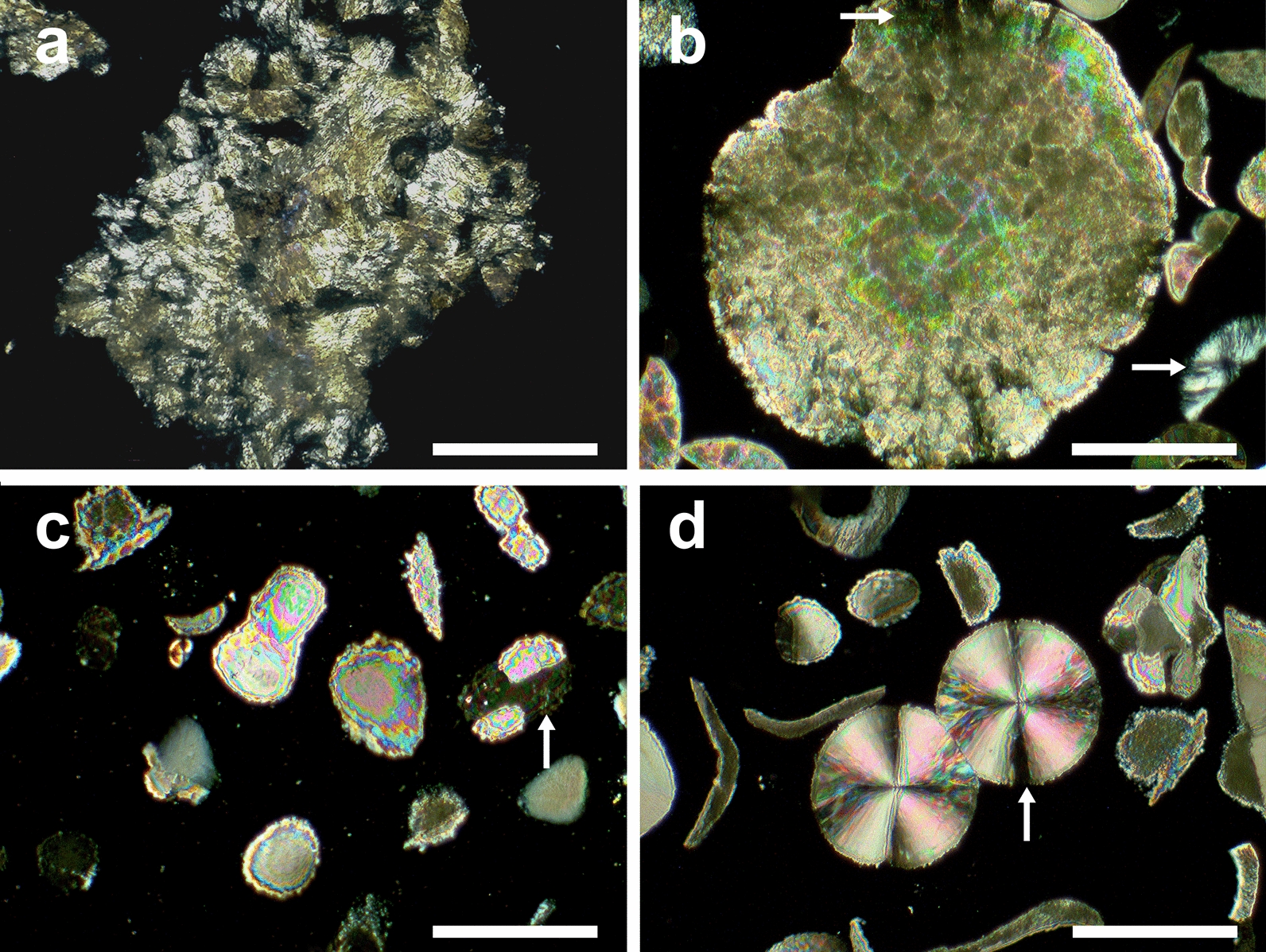
Thin section micrographs of mineral grow composites produced in presence of *Bacillus subtilis* at different condition. **a** BCC-GLY, **b** BCC-GLC, **c** BCC-LAC, and **d** BCC-ACE. Images were acquired with polarized and analyzed light. They show size, shape, and optical properties of biogenetic crystals from diverse media. Arrows symbol show a key optical property, of light extinction (see result and discussion text). Scale bar: 100 μm

A petrographic study was performed in mineralized colonies of *B. subtilis* grown on different media (Fig. 2). The mineral shape, size composite, internal structure, and optical properties were shown in polarizing and analyzed light, where the black background (epoxy resin, Additional file 1: Fig. S2) is surrounding Biogenic Carbonate Crystals (BCC) in bright colors. In GLY condition (Fig. 2a), BBC shown the most amorphous growth habit. All BCC shown at least three interference colors of 4^th^ order in Michel-Lévy scale with wedge-shaped margin, suggesting calcite presence. Moreover, in BCC-LAC and BCC-ACE (Fig. 2c, d), it is clear light extinction North–South/Este-West between microscope polarizing axes and mineral axes (Maltese cross), which fits to fiber-radial crystal distribution. BCC-GLC (Fig. 2b) exhibited a better spherical and bigger size composite (~ 300 µm of diameter). Nevertheless, also it can be overbed ring growths of smaller crystals. In all conditions, BCC apparently grown in a layered crystal arrange, although in BCC-GLY (Fig. 2a) mineral layer seems do not have an order in comparison to other obtained crystals. BCC-GLC (Fig. 2b) have a crystal layers starting in several nuclei all over the colony. It can be observed as changes in color separated by color lines. However, for BCC-LAC (Fig. 2c) and BCC-ACE (Fig. 2d), layers start from top to the bottom layer, in that order until forms the crystal. Finally, micrite present botryoidal growth with nodular arrangement in BCC-GLC (Fig. 2b and Additional file 1: Fig. S3).

### BCC crystalline properties

**Fig. 3:**
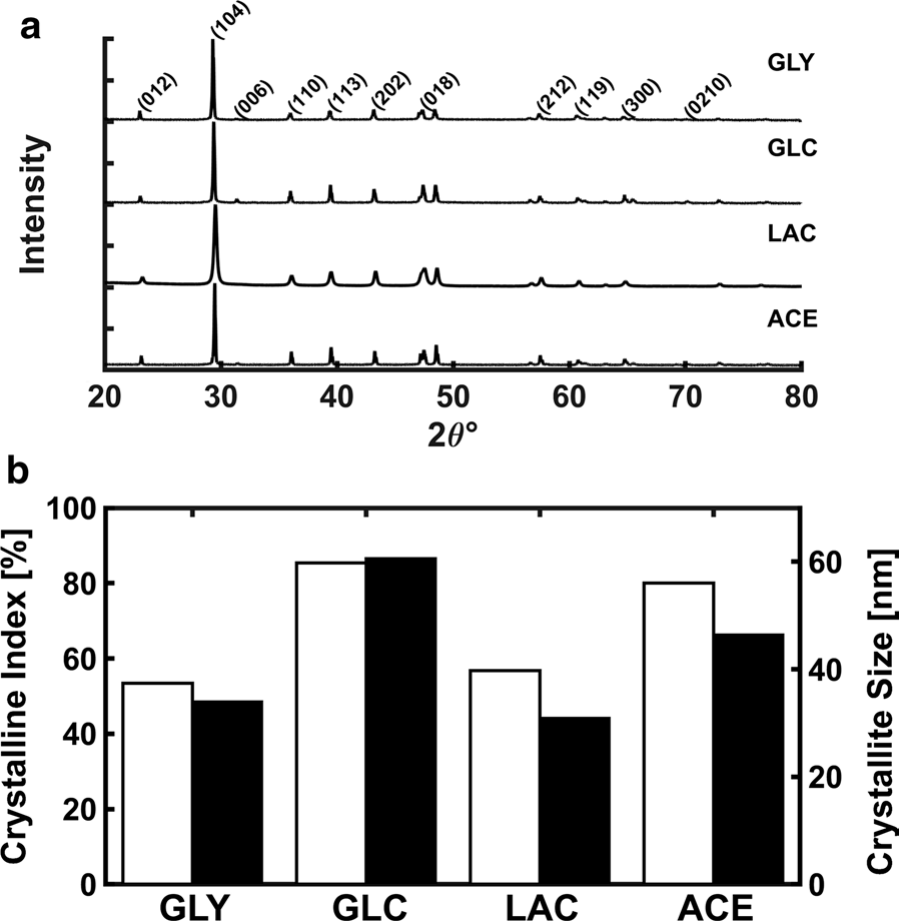
Crystallographic properties of BCC. Diffractogram **a** of BCC-GLY, BCC-GLC, BCC-LAC and BCC-ACE. **b** Bars show the crystallinity index (white, left scale) and crystallite size of 104 peak (black, right scale) of composites obtained in each condition

To determine the crystallographic properties of the BCC produced, the samples were subjected to X-ray diffraction by powder method. Calcite was the only CaCO_3_ polymorphism obtained in BCC of each condition (Fig. 3a), as was inferred from the intensity of 104 surface plane. The BCC-GLC shown the highest crystallinity (85%), followed by BCC-ACE (80%), BCC-LAC (56.7%) and BCC-GLY (53.4%) (Fig. 3b). The crystallite size interval for all BCC was between 30 and 60 nm, where the largest and smallest one was obtained in BCC-GLC and BCC-LAC, respectively.

### Crystalline structures and superficial morphology of BCC

The BCC morphology was analyzed by SEM. BCC aggregates sizes varied in the four growth conditions as follow: 33–217 μm of BCC-GLY; 43–316 μm of BCC-GLC; 48–118 μm of BCC-LAC and 38–172 μm of BCC-ACE.

**Fig. 4:**
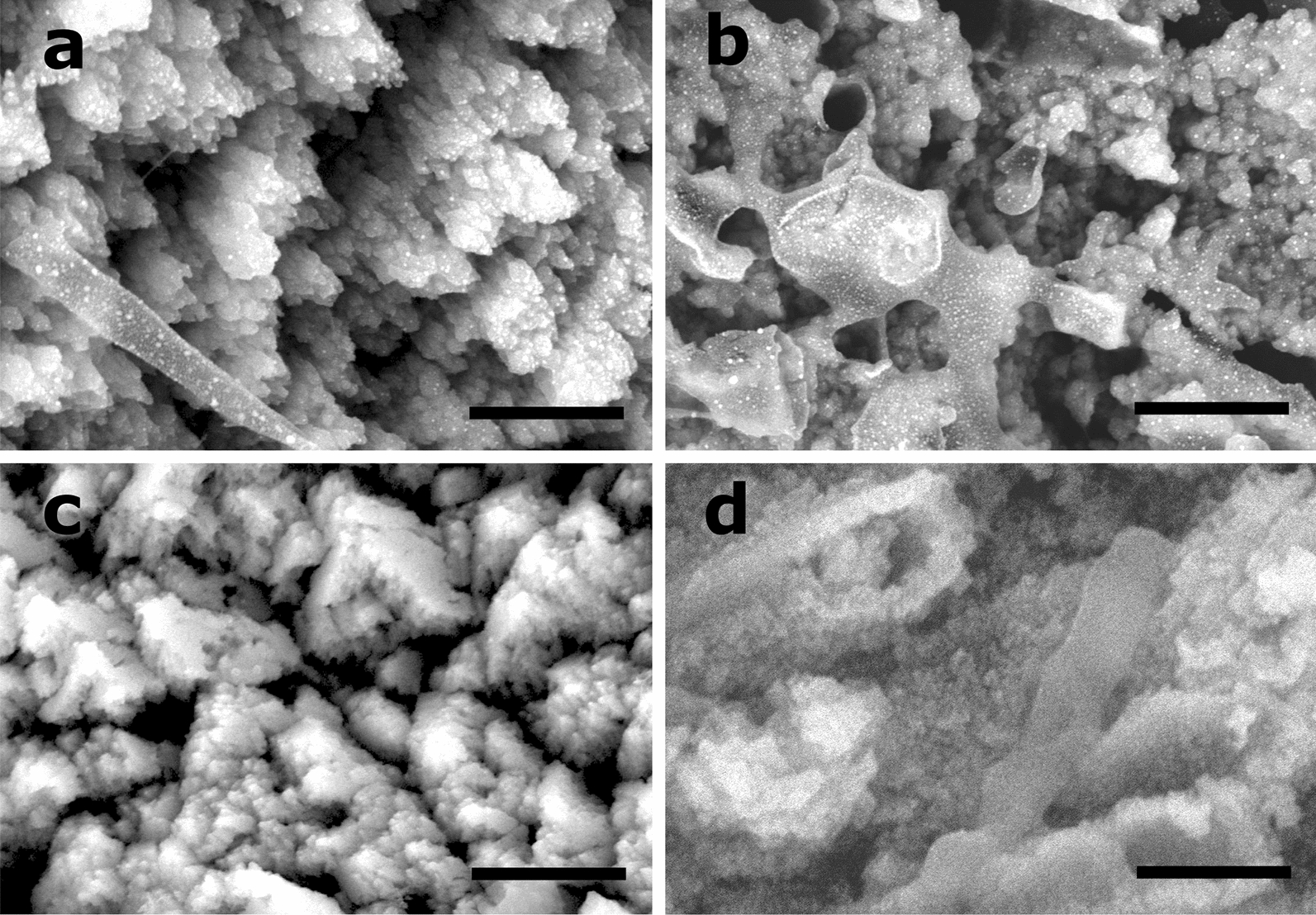
BCC structures formed in non-coupled and coupled calcium-carbon sources. SEM images of BCC-GLC (**a**, **c**) and BCC-ACE (**c**, **d**) are shown. Scales (**a**) and rhombohedral calcite irregular shapes (**c**) are present. Crystallites grow over calcite aggregates and mineralized EPS in non-coupled sources (**a**, **b**). Besides, induced mineralization occurs over the cell wall in coupled source (**d**). Scale bars: 1 μm.

**Fig. 5:**
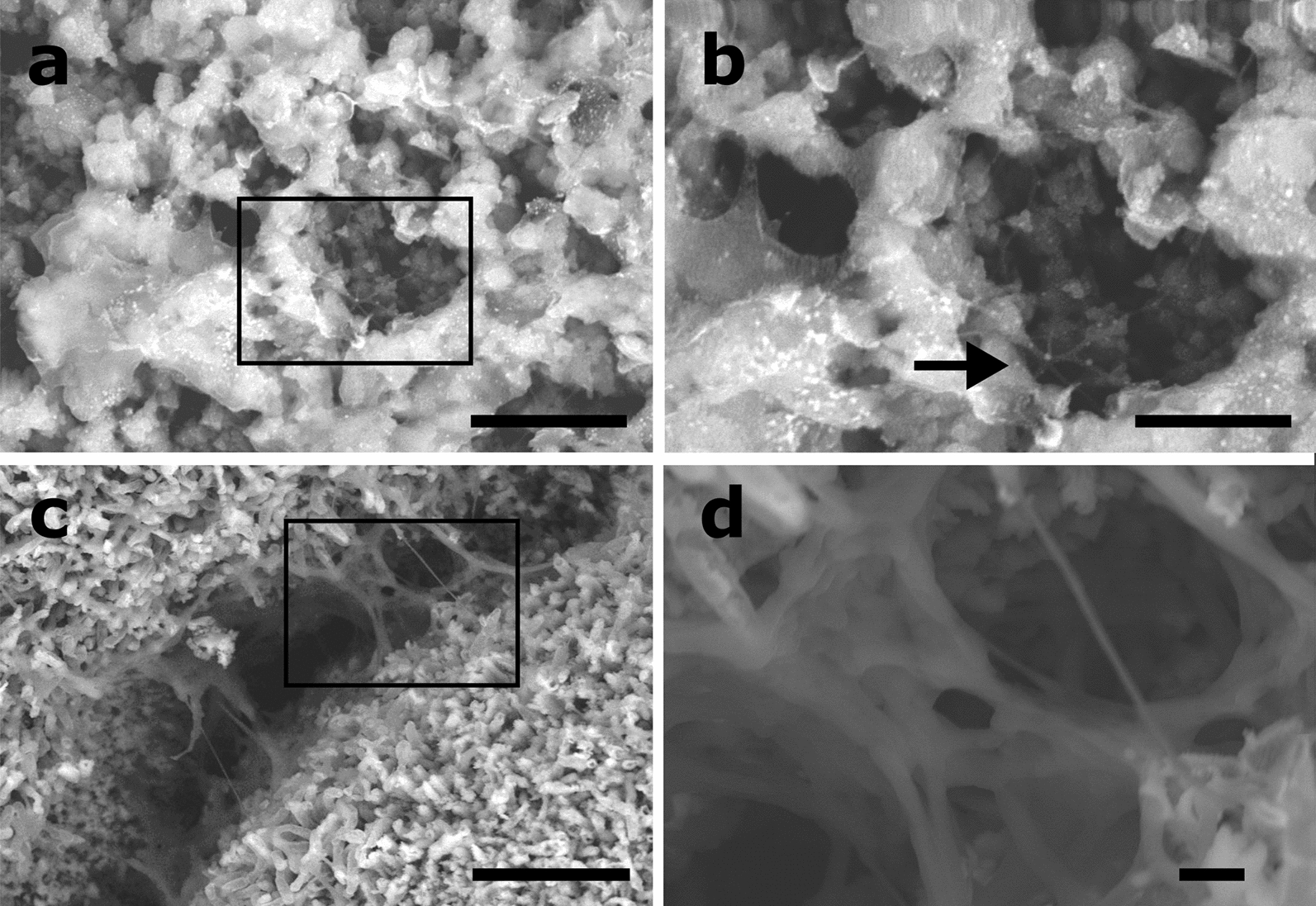
Mineralized micro and nanofibers of BCC-EPS. SEM images of **a** BBC-GLC and **c** BCC-LAC are shown. The rectangles (**b**, **d**) indicate its respective magnifications, and nanofibers are pointed out (black arrow). Scale bars: 10 µm (**a**) and 1 µm (**b**), and 1 µm (**c**) and 0.5 µm (**d**)

BCC presented different mineralized structures, such as scales (Fig. 4a) or rhombohedral irregular shapes (Fig. 4c) formed in uncouple and couple calcium-carbon sources, respectively. Those structures correspond to calcite morphology. The mineralized EPS were more evident in BCC-GLY and BCC-GLC (Fig. 4b) than BCC-LAC or BCC-ACE. Crystallite formations were evident just in non-coupled sources (Fig. 4a, b and Additional file 1: Fig. S4a, b); their sizes are in concordance to XRD data (Fig. 3b). In all condition cells were mineralized, INDBM was evident in coupled calcium-carbon sources, where rhombohedral shapes occurred in cell wall (Fig. 4d). The mineralized cells were also observed inside colonies formed in ACE condition (Additional file 1: Fig. S5). As well, other ultra-structures were observed between mineralized cells, like fibers growing in nets (0.5 μm) between foliated scales and mineralized EPS of BCC-GLC (Fig. 5a) or forming bridges (10 μm) between colonies in BCC-LAC (Fig. 5a).

### BCC composition analysis

**Fig. 6:**
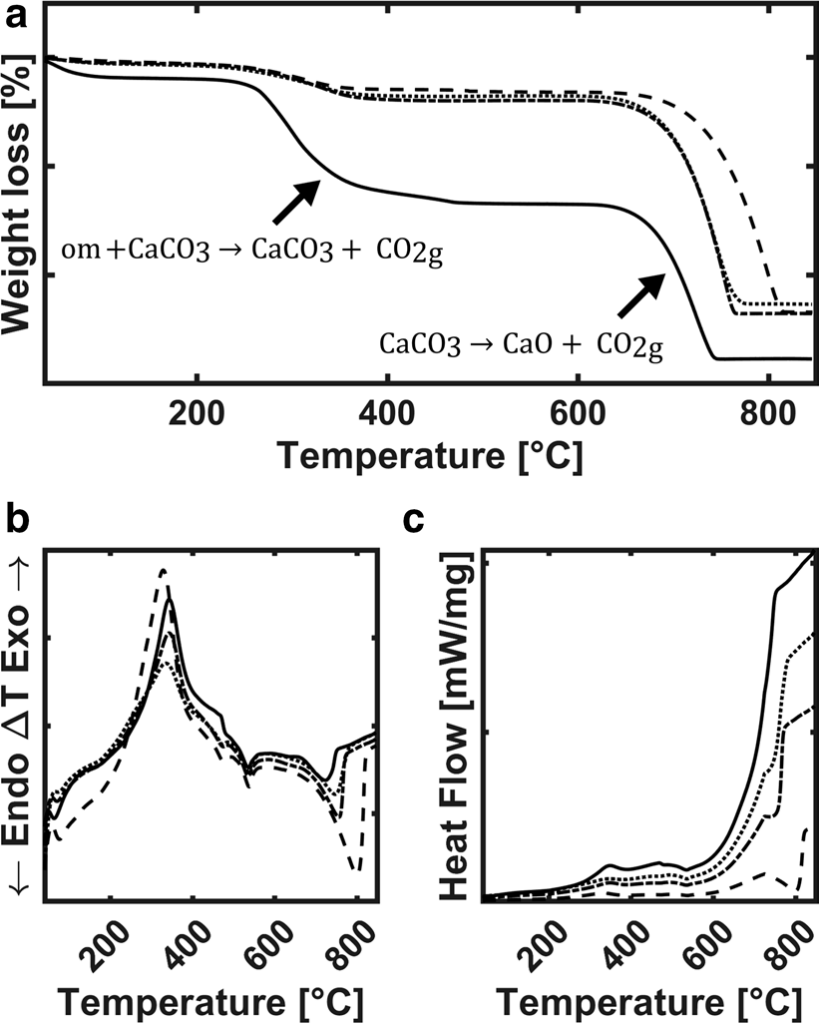
Phase transition of composites formed on different media. **a** Thermogravimetric (TGA) profile and material weight loss of organic matrix (om), carbon dioxide (CO_2_) and calcium oxide (CaO). **b** Differential thermal analysis and **c** differentia scanning calorimetry (DSC) show degradation enthalpy and crystalline transition of amorphous calcium carbonate (ACC) to crystalline CaCO_3_. Lines indicate BCC-GLY (solid), BCC-GLC (dot), BCC-LAC (grid), and BCC-ACE (dashed)

The thermogravimetric analysis (TGA) of BCC showed similar patterns in all four conditions (Fig. 6). They presented three important mass lost (Fig. 6a) at 75 °C and 237 °C and between 700 and 800 °C. These changes are more pronounced in BCC-GLY. The first one could be associate to dehydration of amorphous CaCO_3_. Second one corresponds to organic matter loss, where BCC-GLY reduced its mass 22%, BCC-LAC 6.5%, BCC-GLC 5.5%, and BCC-ACE 5%. The last process belongs to CaCO_3_ decomposition to CaO on BCC, where in BCC-GLC, BCC-LAC and BCC-ACE the lost occurred between 38.3 and 40.4%. While in BCC-GLY weight lost was 28.8%. These thermic BCC processes (Fig. 6b) could be described as: endothermic one near 100 °C, followed by exothermic one between 335 and 340 °C, and two endothermic process at 535 °C and between 728 and 802 °C. The changes in heat flow during thermal decomposition (Fig. 6c) were measured by enthalpy (ΔH). In 331–338 °C the values were 430.96, 480.73, 579.69, 10,497 J g^−1^ °C^−1^, with peak temperature in 332, 350, 347.5 and 349 °C for BCC-ACE, BCC-LCA, BCC-GLC, and BCC-GLY, respectively. In 744–800 °C the reported values were 1149.6, 1758.8, 2275.6, 4487.5 J g^−1^ °C^−1^ at 744.38, 745.74, 755.64, 799.99 °C for BCC-GLY, BCC-GLC, BCC-LAC, and BCC-ACE, respectively. The BCC with better and worst thermal stability were obtained in ACE and GLY media, respectively. All data above indicate the presence of higher organic matter in BCC-GLY.

Additionally, EDX analysis of BCC surfaces showed that all composites were mainly formed by C, O, Ca and, in lower proportion by N, P, and S (Additional file 1: Fig. S6a). Besides, all BCC were evaluated by FTIR-ATR spectrophotometry in the near infrared spectrum. The absorption bans of CO_3_^2−^-calcite vibrational groups were observed (Additional file 1: Fig. S6b), such as ν_4s_CO_3_, at 712 cm^−1^, ν_2as_CO_3_ at 870 cm^−1^ and ν_3as_CO_3_ at 1409 cm^−1^ (Plav et al. 1999). Moreover, the presence of functional groups of organic origin was found, such as ν_s3_ PO_4_ at 580 cm^−1^ and ν COP at 1145 cm^−1^; narrowing of –C–O–C– and C–O related to polysaccharides between 1000 and 1154 cm^−1^; amide and amino groups as νs NH_2_ at 1623 cm^−1^; ν_s_ CO at 1653 cm^−1^; ν_s_ CO^free^ at 1682 cm^−1^; δNH at 776 cm^−1^ an sulfur group as ν_as_ SO at 1390 cm^−1^. After 1800 cm^−1^, no signal of a functional group was observed.

## Discussion

The BCC produced in presence of *B. subtilis* had different morphological and crystallographic properties, depending of media composition: (a) conditions where the calcium source is not associated to carbon one, non-coupled (GYL and GLC); or (b) where the calcium is attached to the carbon source, coupled (LAC and ACE). The predominant morphologies in petri dish were mineralized colonies with rounded shape and convex (Fig. 1), in contrast to experiments done in liquid media were dumbbell, rhombohedral and spherulitic shapes are common (Han et al. 2019). Independently to media, the main factor that induced carbonate precipitation is the microenvironment alkalinization, that enhance complexation of Ca^2+^ free ions with free CO_3_^2−^ ion (Dupraz et al. 2009). Specifically, in nutrient media, glutamine deamination (Dervaux et al. 2014), and carbonic anhydrase activity (Frankel and Bazylinski 2003; Perito and Mastromei 2011; Oppenheimer-Shaanan et al. 2016; Han et al. 2019) could provoke this pH changes (Jiménez-Delgadillo et al. 2018). pH changes in long term *Bacillus* culture in agar-rich media (Robinson et al. 1991).

Only BCC-GLC were orange pigmented (Fig. 1b), which is related to iron chelation by pulcherriminic acid produced in biofilms of carbohydrates supplemented media (Arnaouteli et al. 2019).

Thin section micrography show birefringence phenomena under polarized light (Fig. 2). However, due the presence of optical interference color of light green-extinction characteristic 4th order birefringence it can be interrelated has arrangement of typical calcite carbonate crystals (Aizenberg and Hendler 2004). However, did not show the characteristic calcite cleavage. Difference in the interference color in crystals indicates a non-oriented arrangement of microcrystal growth, it can be produced by the randomized growth of bacteria colonies. However, mineral growth in glycerol media is also determined by crystal nucleation in EPS produced by cells (Oppenheimer-Shaanan et al. 2016), that is probably the cause of its amorphous habit. Micrite is common in marine sediments and microbialites (Perri and Spadafora 2011) with spherical and radial-fibers growth as we observed in all BCC; mineralized cells are also present (Rasmussen and Muhling 2019).

Several strains of *Bacillus* could mineralize different polymorphisms of CaCO_3_, such calcite and vaterite (Seifan et al. 2016; Andrei et al. 2017; Huynh et al. 2018), but factors that induce only one kind of polymorphism are not jet understood. In this work well only observe calcite production, even if calcium-carbon source was non-coupled. Other authors have identified this crystalline phase in *B. subtilis* (Zhuang et al. 2018; Han et al. 2019). The obtained BCC had different crystallinity (BCC-GLY < BCC-ACE < BCC-LAC < BCC-GLC), possibly related to mineralization process in each formation-condition (Fig. 3b). The IC of BCC-GLC may be similar to abiotic process pH, Ca^2+^ concentration and nucleation sites are critical (Perito and Mastromei 2011). For coupled calcium-carbon sources, like ACE (Fig. 4d, Additional file 1: Figure S5), the mineralization mainly occurred over cellular structures such as cell wall and EPS (Marvasi et al. 2012; Dhami et al. 2013). In that sources, critical Ca^2+^ concentration could be accumulated in cell wall because the efflux pump and specific channels (Saier et al. 2002). The membrane and cell wall components could be nucleation sites because its negative charges of teichoic acid that attract Ca^2+^ (Perito et al. 2014).

Lower IC in BCC-LAC and BCC-GLY could be associated to higher production of organic matter or amorphous calcium carbonate (ACC). This is the polymorphic precursor of crystalline structures under biotic or abiotic mineralization (Bots et al. 2012; Cantaert et al. 2016). Some interaction with glycoproteins or organic molecules increased the ACC stability and prevent spontaneous crystallization (Aizenberg et al. 2002; Weiner et al. 2003). Additionally, in non-coupled calcium-carbon sources (GLY and GLC), BCC nanodeposits with the same sizes of calculate crystallite were evident over calcite scales and mineralized EPS (Fig. 4a, b, Additional file 1: Fig. S4). This is the first work where such kind of precipitants are observed in biocomposites of *B. subtilis*.

The main components of *B. subtilis* EPS, that promote calcite aggregation, are TasA and TapA amyloid proteins and exopolysaccharides (Azulay and Chai 2019); moreover, there are reports of nanotubes formation when *B. subtilis* grows in rich media (Bhattacharya et al. 2019). In this study, both structures were observed in BCC-GLC (Fig. 5b) and BCC-LAC (Fig. 5c), respectively.

In order to understand the BCC composition, TGA and DSC analysis were performed (Fig. 6). Thermic change of biotic and abiotic vaterite was reported with an exothermic peak between 317 and 318 °C, that was associated to organic matter decomposition with CO_2_ and NO_2_ release (Rodriguez-Navarro et al. 2007). Furthermore, at 700–800 °C a second reaction occurs, from CaCO_3_ to CaO (Al Omari et al. 2016). As we saw, BCC-GLY presented greater abundance of organic matrix; however, temperature decomposition not follow enthalpy rise. Some organic acids could change temperature peaks of that process (Li et al. 2017).

Mineralization degree (BCC-ACE > BCC-LAC > BCC-GLC > BCC-GLY) also affected thermic stability of BCC in relation to enthalpy and decomposition temperatures (Fig. 6). This may be to mineral abundance or lattice variation (Pokroy et al. 2006), such as in BCC-GLC with higher crystallinity but low presence of CaCO_3_.

In BCC, EPS could increase complexation by the interaction of the ions with the electronegativity charge of its expose functional groups (Ercole et al. 2007; Oppenheimer-Shaanan et al. 2016). This explain the random nucleation of minerals that occurs in the bacteria microenvironment. However, in BCC-GLY the synthesis level of EPS increases the amount of nucleation sites but affected negatively the crystallinity. EPS have a key role in CaCO_3_ precipitation (Arias and Fernández 2008), and *B. subtilis* produce high amount of different types in glycerol supplemented media (Oppenheimer-Shaanan et al. 2016). Besides, EPS is important to CaCO_3_ precipitation (Decho 2010; López-Moreno et al. 2014), because it attract Ca^2+^ to its chemical functional groups, such as: COO–, NH_3_–, PO_4_– and SO_4_– groups (Schmitt and Flemming 1998; Humbert and Quilès 2011). Those group, related to main EPS components (exopolysaccharides and proteins) play a key role in BCC formation (Azulay and Chai 2019). P content may be related to extracellular DNA usually present in EPS (Peng et al. 2020).

In this study the BCC produced in presence of *B. subtilis* were constituted manly by calcite. We demonstrated that the quality of this biogenic calcite was only influenced by calcium-carbon source. In that sense, the best composites were obtained when bacteria were grown in supplemented medium by glucose and calcium chloride. Finally, this non-coupled calcium-carbon condition promotes several nucleation sites for CaCO_3_ precipitation, and it was no need to provide with urea to achieve the necessary alkalinity.

## Supplementary information

**Additional file 1.** Supplementary material.

## Data Availability

All relevant data are within the manuscript and its Additional file 1.

## Abbreviations

*B. subtilis*: *Bacillus subtilis*
BCC: Biogenic carbonate crystals
GLY: Glycerol + CaCl_2_
GLC: Glucose + CaCl_2_
LAC: Calcium lactate
ACE: Calcium acetate
OM: Organic matter
ACC: Amorphous calcium carbon
CBM: Controlled biological mineralization
INDBM: Induced biological mineralization
INFBM: Influenced biological mineralization
EPS: Extracellular polymeric substances
SEM: Scanning electron microscopy
EDX: Energy dispersive X-ray spectroscopy
XRD: X-ray diffraction
CI: Crystallinity index
ATR-FTIR: Fourier transformed infra-red spectrometry
TGA: Thermogravimetric
DSC: Differentia scanning calorimetry
