# Supplementary material for: Novel method to achieve crystallinity of calcite by Bacillus subtilis in coupled and non-coupled calcium-carbon sources
Source: AMB Express. 2020 Sep 29;10:174. doi: 10.1186/s13568-020-01111-6 (PMC7524977; doi:10.1186/s13568-020-01111-6)
Supplement: Supplementary file 1 — Additional file 1. Supplementary material. [file 13568_2020_1111_MOESM1_ESM.docx]

**Applied Microbiology and Biotechnology Express**

Novel method to achieve crystallinity of calcite by *Bacillus subtilis* in coupled and non-coupled calcium-carbon sources

Ferral-Pérez H.^ab^, Galicia-García M.^b^, Alvarado-Tenorio B.^c^, Izaguirre-Pompa A^d^, Aguirre-Ramírez M.^a^*

^a^ Laboratorio de Biología Celular y Molecular. ^b^ Laboratorio de Electroquímica. ^c^ Laboratorio de Bioquímica Funcional y Proteómica del Estrés. Departamento de Ciencias Químico-Biológicas, Instituto de Ciencias Biomédicas, Universidad Autónoma de Ciudad Juárez, Cd. Juárez, Chih., 32310. México.

^d^ Laboratorio de Geología. Departamento de Ingeniería Civil y Ambiental, Instituto de Ingeniería y Tecnología, Universidad Autónoma de Ciudad Juárez, Cd. Juárez, Chih.

* Corresponding author: Marisela Aguirre-Ramírez, [marisela.aguirre@uacj.mx](mailto:marisela.aguirre@uacj.mx)

**Additional file:**

**Fig S1.** pH values of *B. subtilis* cultures grown in Nutrient Broth supplemented conditions. Data represents the average of three independent experiments ± standard deviation.


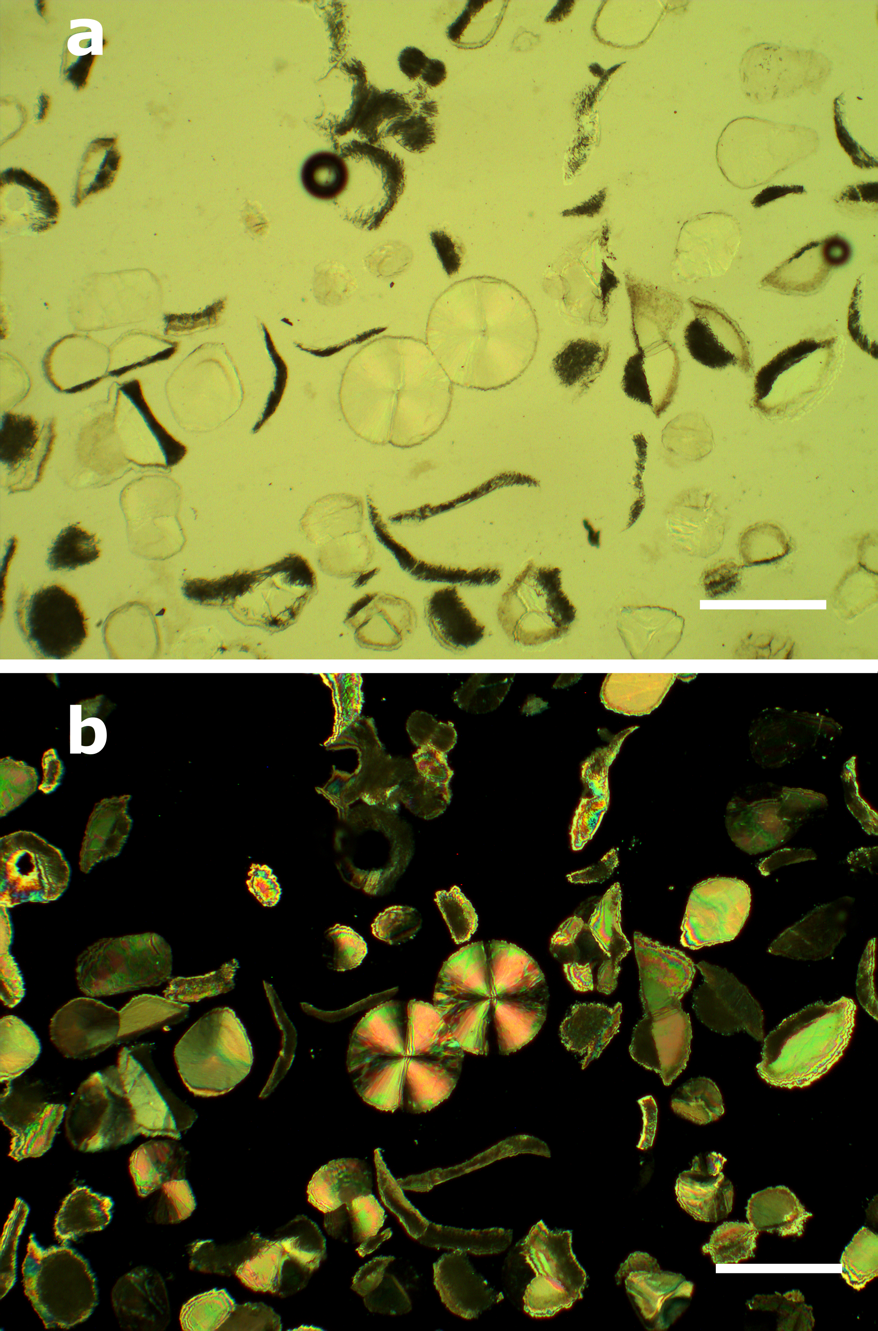


**Fig. S2** Thin section micrograph of BCC-ACE observed under bright field and polarized light. Scale: 100 µm.


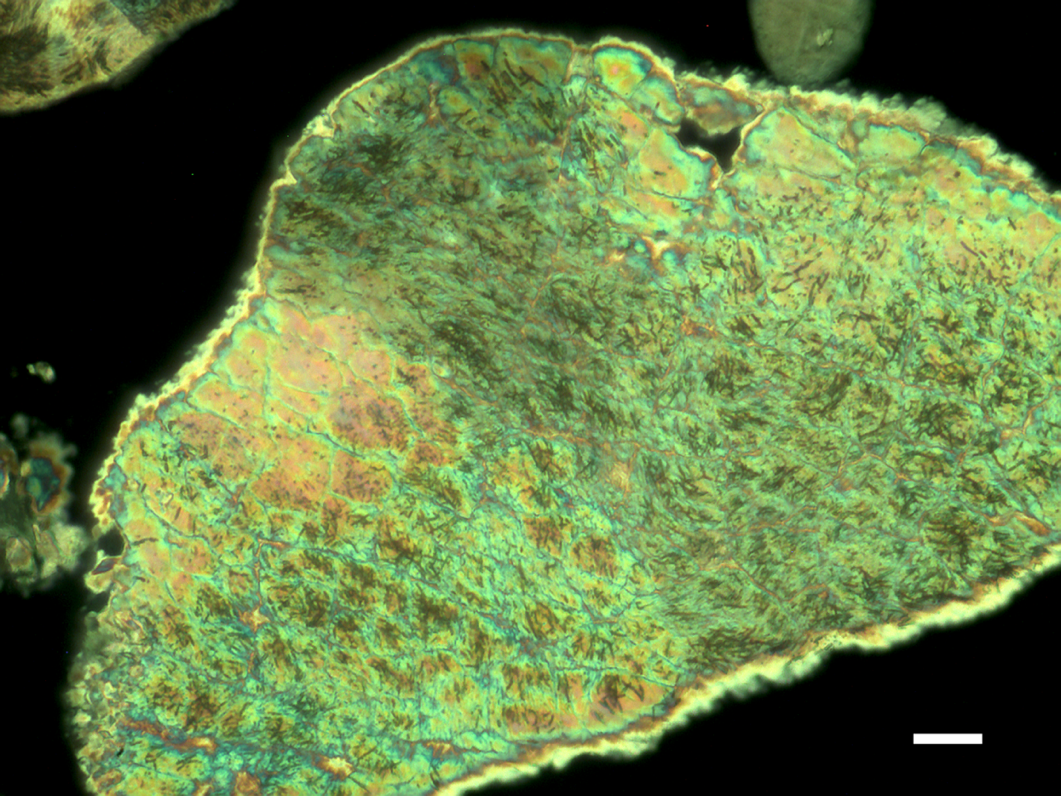


**Fig. S3** Thin section micrograph of BCC-GLC observed under polarized light. Mineral botryoidal growth, triple or double joins in twinned crystal, and baciliar shapes are shown. Scale: 10 µm


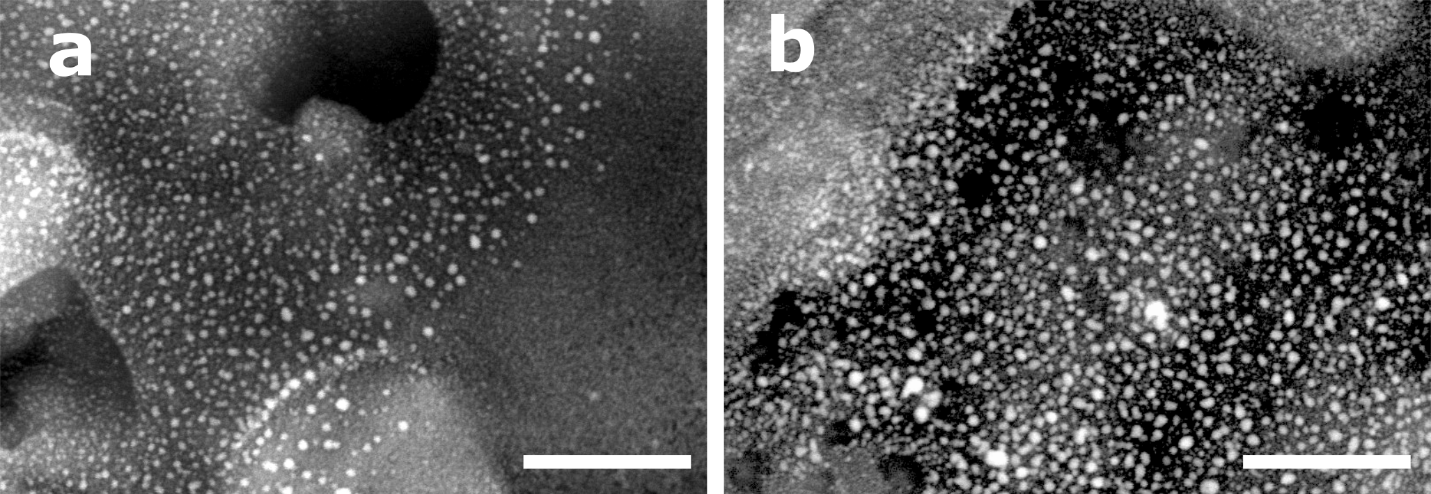


**Fig. S4** SEM images showing the crystallites formed over EPS of BCC-GLY (a) and BCC-GLC (b). Scale: 500 nm.


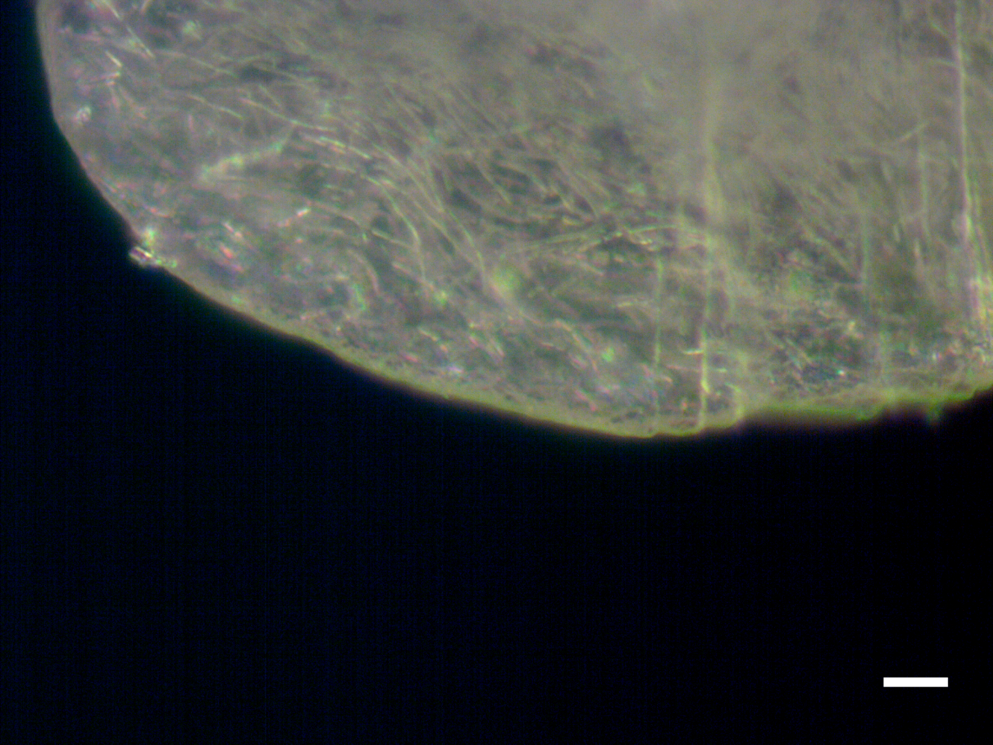


**Fig. S5** Micrograph of BCC formed in ACE conditions. The mineralized cell-filaments are observed. Scale: 10 µm.

.
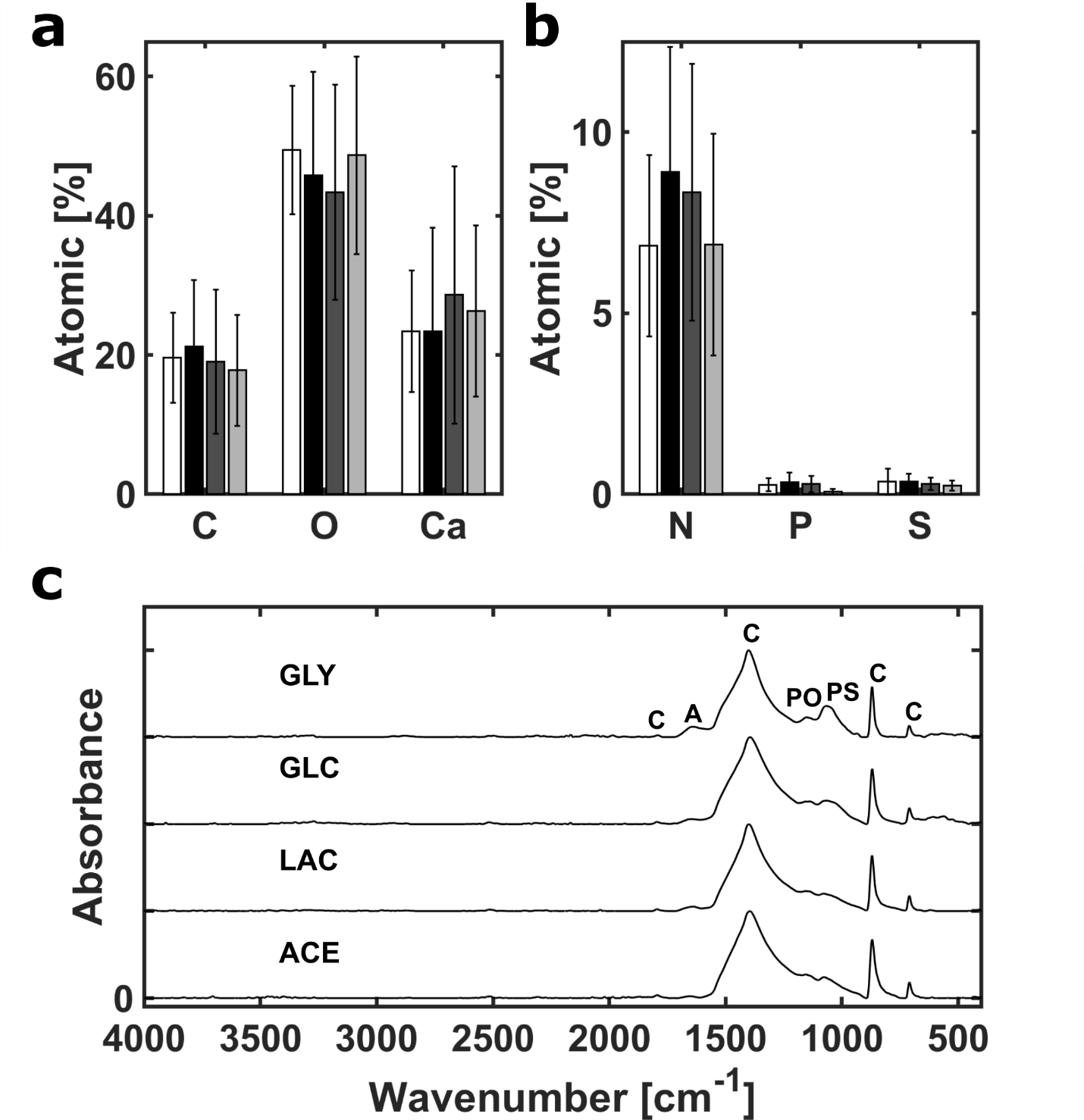


**Fig. S6** BCC composition. (a) Bars correspond to the average of six punctual EDX measurements taken over four different crystals of BCC-GLY (white), BCC-GLC (black), BCC-LAC (dark gray) and BCC-ACE (gray). Error bars indicate standard deviation. (b) FTIR-ATR spectrum of all BCC. The main functional vibrations groups for calcite (C), polysaccharides (PS), phosphates (PO) and amides (A) are marked.
